# Publication of the Journal

**Published:** 1869-06-15

**Authors:** 


### EDITORIAL.

### *Publication of the Journal.*

The recent change in publishers of the JOURNAL, has been necessitated by the removal of the previous publisher from the city. It is practically impossible for the Editors, in the midst of their professional duties, to assume the business management, personally, and find any time to devote to the proper development of the scientific character of this now time-honored organ and exponent of the profession. Under the energetic and experienced control of our new publisher, the JOURNAL has already received a support which will speedily enable us to add largely to its interest and value. Without expressly designating improvements contemplated we promise, in the remaining portion of the year, to furnish our readers a variety and amount of professional reading of the highest character, hitherto unequalled in the history of our magazine.

Since the inauguration of our semi-monthly series the Senior Editor has sustained the largely increased expenditure at great personal pecuniary sacrifice. He has been, and is, the sole proprietor. There remain upon the books thousands of dollars of unpaid subscriptions. Half of what is due would repay him for his advances, and relieve from an incubus which those who know him personally are aware is borne with a patience the farthest possible removed from Job's. We know, now, what makes Bro. Bowling of the Nashville *Journal* one of the most genial and delightful of our contemporaries. Out of the fullness of a joyous heart he exclaims, in a recent number, "This *Journal* owes no man a cent, and no man owes it a cent!" If we did not know Dr. Bowling to be a professional Chevalier Bayard, *sans peur et sans reproche*, we wouldn't believe a word of it.

But this is enough of a highly suggestive topic. In passing we only say: Pay what *thou* owest, so shalt thou rejoice the hearts of editors, publishers and readers, and on January next we shall hope to begin the issue of the JOURNAL once in every seven days, bringing the waters of medical wisdom fresh and sparkling from their original fountains.

